# Biological and molecular profile of fracture non‐union tissue: A systematic review and an update on current insights

**DOI:** 10.1111/jcmm.17096

**Published:** 2022-01-04

**Authors:** Michalis Panteli, James S.H. Vun, Ippokratis Pountos, Anthony J. Howard, Elena Jones, Peter V. Giannoudis

**Affiliations:** ^1^ Academic Department of Trauma & Orthopaedics School of Medicine University of Leeds Leeds UK; ^2^ Leeds Institute of Rheumatic and Musculoskeletal Medicine School of Medicine University of Leeds Leeds UK; ^3^ Leeds Orthopaedic & Trauma Sciences Leeds General Infirmary University of Leeds Leeds UK; ^4^ NIHR Leeds Biomedical Research Unit Chapel Allerton Hospital Leeds UK

**Keywords:** non‐union(s), nonunion(s), fracture, human tissue, mesenchymal stem cell(s), mesenchymal stromal cell(s)

## Abstract

Fracture non‐union represents a common complication, seen in 5%–10% of all acute fractures. Despite the enhancement in scientific understanding and treatment methods, rates of fracture non‐union remain largely unchanged over the years. This systematic review investigates the biological, molecular and genetic profiles of both (i) non‐union tissue and (ii) non–union‐related tissues, and the genetic predisposition to fracture non‐union. This is crucially important as it could facilitate earlier identification and targeted treatment of high‐risk patients, along with improving our understanding on pathophysiology of fracture non‐union. Since this is an update on our previous systematic review, we searched the literature indexed in PubMed Medline; Ovid Medline; Embase; Scopus; Google Scholar; and the Cochrane Library using Medical Subject Heading (MeSH) or Title/Abstract words (non‐union(s), non‐union(s), human, tissue, bone morphogenic protein(s) (BMPs) and MSCs) from August 2014 (date of our previous publication) to 2 October 2021 for non‐union tissue studies, whereas no date restrictions imposed on non–union‐related tissue studies. Inclusion criteria of this systematic review are human studies investigating the characteristics and properties of non‐union tissue and non–union‐related tissues, available in full‐text English language. Limitations of this systematic review are exclusion of animal studies, the heterogeneity in the definition of non‐union and timing of tissue harvest seen in the included studies, and the search term MSC which may result in the exclusion of studies using historical terms such as ‘osteoprogenitors’ and ‘skeletal stem cells’. A total of 24 studies (non‐union tissue: *n* = 10; non–union‐related tissues: *n* = 14) met the inclusion criteria. Soft tissue interposition, bony sclerosis of fracture ends and complete obliteration of medullary canal are commonest macroscopic appearances of non‐unions. Non‐union tissue colour and surrounding fluid are two important characteristics that could be used clinically to distinguish between septic and aseptic non‐unions. Atrophic non‐unions had a predominance of endochondral bone formation and lower cellular density, when compared against hypertrophic non‐unions. Vascular tissues were present in both atrophic and hypertrophic non‐unions, with no difference in vessel density between the two. Studies have found non‐union tissue to contain biologically active MSCs with potential for osteoblastic, chondrogenic and adipogenic differentiation. Proliferative capacity of non‐union tissue MSCs was comparable to that of bone marrow MSCs. Rates of cell senescence of non‐union tissue remain inconclusive and require further investigation. There was a lower BMP expression in non‐union site and absent in the extracellular matrix, with no difference observed between atrophic and hypertrophic non‐unions. The reduced BMP‐7 gene expression and elevated levels of its inhibitors (Chordin, Noggin and Gremlin) could potentially explain impaired bone healing observed in non‐union MSCs. Expression of Dkk‐1 in osteogenic medium was higher in non‐union MSCs. Numerous genetic polymorphisms associated with fracture non‐union have been identified, with some involving the BMP and MMP pathways. Further research is required on determining the sensitivity and specificity of molecular and genetic profiling of relevant tissues as a potential screening biomarker for fracture non‐unions.

## 1 INTRODUCTION

Bone healing is a complex biological process aiming at restoring the affected area to its pre‐injury levels. This is achieved through repair and regeneration of the cellular and extracellular components, regaining its former biochemical and biomechanical properties.^1^, ^2^ Successful bone healing requires the orchestrated interaction between the biological (cellular, signalling molecules and extracellular matrix) and mechanical environments.^3^ Moreover, according to the ‘Diamond Concept’, other parameters that are considered essential for a successful healing include the local vascularity and the patient's biological fitness and comorbidities.^4^

The definition of non‐union has been inconsistent in the literature. The FDA (Food and Drug Administration), however, defines non‐union as incomplete fracture healing within 9 months following injury, coupled by the lack of progression in radiological signs of healing over the course of three consecutive months.^5^ Despite the advancement in both the understanding of fracture healing and some of the pathways that regulate it, the rates of fracture non‐union remain largely unchanged over the years. To date, fracture non‐union remains common, occurring in 5%–10% of the 850,000 fractures seen yearly in the UK.^6^ This poses a significant direct and indirect socioeconomic burden through prolonged medical treatments and productivity losses.^6^ Further understanding of the biological processes and underlying mechanisms, along with their interactions, leading to fracture non‐union need to be elucidated in order to reduce this risk.

We have previously published a systematic review outlining the biological and molecular profile of ‘non‐union tissue’.^1^ Nevertheless, one critically relevant and important aspect not previously considered because of the scarce evidence at the time was the relevance of tissues harvested from sites away from the non‐union site, such as peripheral blood and bone marrow products. Moreover, the accelerated improvement in laboratory techniques over the last decade also meant the biological and molecular understanding of the multiple pathways involved in bone healing is everchanging. Consequently, the herein study provides an up‐to‐date review on the knowledge that has been acquired in this important clinical condition. We aim to summarize the current evidence on (i) macroscopic and microscopic characteristics; (ii) cellular characteristics and function (cell surface protein expression, morphology, viability, proliferation, senescence, mineralization and alkaline phosphatase [ALP] activity); (iii) molecular characteristics (protein, mRNA, miRNA and gene expression) of non‐union tissue and relevant tissues; (iv) differences between atrophic and hypertrophic non‐unions; (v) effect of intervention(s) on non‐union tissue and relevant tissues; and (vi) genetic predispositions to fracture non‐union.

## 2 MATERIALS AND METHODS

This systematic review was conducted according to the PRISMA guidelines.^7^ Our protocol was similar to that of our previous publication, with the only difference being the addition of other types of tissues not harvested from the non‐union site (‘relevant tissue’) in our inclusion criteria.^1^ We define ‘relevant tissue’, as bone marrow or peripheral blood derived products, investigated to identify associations with progression to non‐union. The reason for including studies assessing relevant tissue was due to the growing body of evidence demonstrating the correlation of these tissues with the occurrence of non‐union, which we feel could be helpful to guide clinicians in their practice.

### 2.1 Eligibility criteria

The inclusion criteria were as follows: (i) tissue obtained from the non‐union site and processed for defining its characteristics and properties, OR studies assessing tissue relevant to non‐union as defined above (‘relevant tissue’); (ii) only tissue acquired from human subjects was included; (iii) articles were published in English language; (iv) the full text of each article was available; and (vi) for non‐union tissue, articles published between August 2014 (date of our previous publication) and 2 October 2021; for relevant tissue, no publication date restrictions were imposed. Studies that did not fulfil the eligibility criteria were excluded from further analysis.

### 2.2 Search strategy and information sources

Adhering to our previously published protocol, the following databases were used during literature search: PubMed Medline; Ovid Medline; Embase; Scopus; Google Scholar; and the Cochrane Library. The full search strategy is as detailed in Table 1. Briefly, the search terms included non‐union(s), nonunion(s), human, tissue, bone morphogenic protein(s) (BMPs) and MSCs. Bibliographies of all identified articles were collected in Endnote X9, manually reviewed and searched for any potentially eligible studies.

**TABLE 1:** PubMed search strategy (searched 2 October 2021)

1.	(("non‐union"[All Fields] OR ("nonunion"[All Fields] OR "nonunions"[All Fields]))
2.	("mesenchymal stem cells"[MeSH Terms] OR ("mesenchymal"[All Fields] AND "stem"[All Fields] AND "cells"[All Fields]) OR "mesenchymal stem cells"[All Fields] OR ("mesenchymal"[All Fields] AND "stem"[All Fields] AND "cell"[All Fields]) OR "mesenchymal stem cell"[All Fields]
3	"MSC"[All Fields]
4.	("mesenchymal stem cells"[MeSH Terms] OR ("mesenchymal"[All Fields] AND "stem"[All Fields] AND "cells"[All Fields]) OR "mesenchymal stem cells"[All Fields] OR ("mesenchymal"[All Fields] AND "stromal"[All Fields] AND "cell"[All Fields]) OR "mesenchymal stromal cell"[All Fields])
5.	"bone morphogenetic proteins"[MeSH Terms] OR ("bone"[All Fields] AND "morphogenetic"[All Fields] AND "proteins"[All Fields]) OR "bone morphogenetic proteins"[All Fields] OR ("bone"[All Fields] AND "morphogenetic"[All Fields] AND "protein"[All Fields]) OR "bone morphogenetic protein"[All Fields]
6.	("tissue s"[All Fields] OR "tissues"[MeSH Terms] OR "tissues"[All Fields] OR "tissue"[All Fields])))
7.	(humans[Filter])
8.	(english[Filter]))
9.	2 OR 3 OR 4 OR 5 OR 6
10.	1 AND 9
11.	10 AND 7 AND 8

### 2.3 Study selection

Two of the authors (MP and JV) performed the eligibility assessment independently, in an unblinded, standardized manner. Title and abstract sift were conducted first, followed by review of full text by MP and JV. Only studies fulfilling the eligibility criteria were included. Data of each eligible study were independently extracted by MP and JV, with results checked by the third author (IP). Any disagreement between reviewers was resolved by consensus, and if necessary, the senior researcher (PVG) was consulted.

### 2.4 Extraction of data

Information on author, year of publication, patient demographics, non‐union site, the duration and type of non‐union, characteristics of non‐union tissue (macroscopic/microscopic), cellular characteristics and functions (cell surface protein expression, morphology, viability, proliferation and cellular senescence), molecular characteristics (gene expression, protein expression) and effect of additional interventions were all carefully extracted.

### 2.5 Data analysis

Outcomes of interest as mentioned in ‘Extraction of data’ section were inserted in an electronic database. Wherever possible, each characteristic of tissue samples was compared across different studies. We also evaluated the effect of any interventions documented in these studies. Qualitative results were summarized and presented in tables, whereas quantitative results are presented with *p* values if stated by the study. Statistical comparison was not made between studies, due to the heterogeneity in terms of study methodologies observed in each of these *in vitro* studies.

## 3 RESULTS

### 3.1 Literature search

**FIGURE 1:**
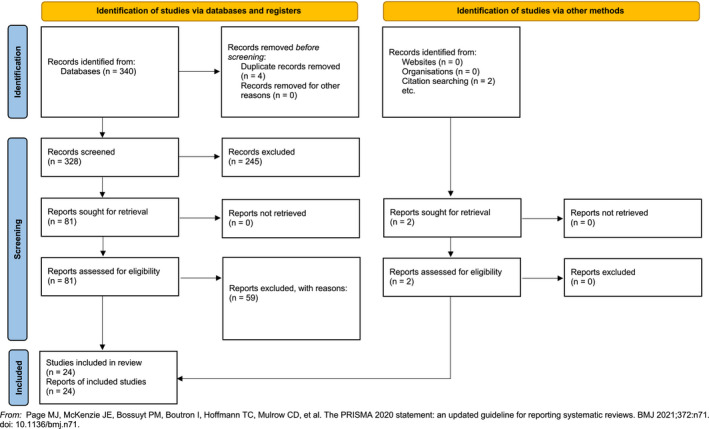
PRISMA 2020 flow diagram—study selection

The electronic literature search retrieved 342 citations, of which 24 met the inclusion criteria for the final analysis (Figure 1).^8^, ^9^, ^10^, ^11^, ^12^, ^13^, ^14^, ^15^, ^16^, ^17^, ^18^, ^19^, ^20^, ^21^, ^22^, ^23^, ^24^, ^25^, ^26^, ^27^, ^28^, ^29^, ^30^, ^31^ Overall, 10 studies^8^, ^9^, ^10^, ^11^, ^12^, ^13^, ^14^, ^15^, ^16^, ^17^ assessed non‐union tissue (Table 2), whereas 14 studies^18^, ^19^, ^20^, ^21^, ^22^, ^23^, ^24^, ^25^, ^26^, ^27^, ^28^, ^29^, ^30^, ^31^ investigated relevant tissue (Table 3).

**TABLE 2:** Non‐union tissue: patient demographics

Author	Year	Time frame	Number of specimens	Site of non‐union	Patients’ age (mean ± SD)	Amount of tissue
Cuthbert^8^	2020	Not mentioned	Atrophic non‐union: 20 (11 males); critical size defects requiring induced membrane/Masquelet procedure: 15 (10 males); BMA: 8 (3 males)	Not mentioned	Atrophic non‐union: median age 53, range 23–81; critical size defects requiring induced membrane/Masquelet procedure: median age 61, range 19–80; BMA: median age 38, range 19–52	Atrophic non‐union: not mentioned; induced periosteum: 1 cm of membrane tissue from centre of bone defect area; BMA: not mentioned
Wei^9^	2020	Not mentioned	Atrophic non‐union: *n* = 3; controls (healed fractures): *n* = 3	Not mentioned	Not mentioned	Not mentioned
Wang^10^	2018	Not mentioned	8 non‐unions compared to 8 with uneventful healing	Not mentioned	Not mentioned	Not mentioned
Vallim^11^	2018	Not mentioned	15 (9 male)	Tibia: 3; femur: 4; humerus: 7; ulna: 1	46.4 ± 12.5	Approximately 1 cm^3^
Takahara^12^	2016	Not mentioned	4 (2 male)	Femur: 1; humerus: 2; clavicle: 1	65.3 ± 5.4	"Small amount"
Schira^13^	2015	Not mentioned	80 (77 male)	Scaphoid	24.6 years (range, 18–71 years)	Not mentioned
Han^14^	2015	2009 to 2010	11	Not mentioned	40 years (range 27–81 years)	Not mentioned
Wang^15^	2014	October 2010 to March 2014	Hypertrophic non‐union: 20 (15 male); atrophic non‐union: 20 (14 male)	Hypertrophic non‐unions: femur 8; femoral neck 1; tibia: 2; humerus: 9. Atrophic non‐unions: femur 5; tibia: 8; humerus: 7.	Hypertrophic non‐unions: 39.35 ± 11.67 years Atrophic non‐unions: 33.75 ± 8.37 years	Not mentioned
Schwabe^16^	2014	Not mentioned	Atrophic non‐union: 44 (22 male) (Histology: 25; GF‐quantification: 19); healed fracture: 13 (7 male) (Histology: 5; GF‐quantification: 8)	Non‐union: Femur: 16; tibia; 12; clavicle: 9; ulna: 4; humerus: 3. Control group: tibia: 4; ulna: 4; femur: 2; radius: 1; metacarpus: 1	49 years (range 20–74 years)	Not mentioned
Ismail^17^	2013	Not mentioned	5 (5 male)	Tibia: 1; femur: 3; humerus: 1	27.40 years ± 7.64 (range, 18–17 years)	10 mls of BMA

Abbreviation: BMA, bone marrow aspirate.

**TABLE 3:** Relevant tissue: Patient Demographics.

Author	Year	Time frame	Number of specimens	Site of non‐union	Patients’ age (mean ± SD)	Amount of tissue
Burska^18^	2020	Not mentioned	15 (study group ‐ 10 union; 5 non‐union); 18 (healthy controls)	Femur, tibia	15 (study group ‐ 10 union; 5 non‐union; range 18–70 years); 18 (healthy controls; range 26–64 years)	Not mentioned
El‐Jawhari^19^	2019	Not mentioned	71 (46 male)	Femur, tibia, humerus	Non‐union group: 49 years (range: 18–76); union group: 44 years (range: 20–75); healthy controls: 42 years (range: 23–60)	BMA: 15mls from ASIS; peripheral venous blood: 12mls; serum from healthy controls: not stated
Ouyang^20^	2019	Not mentioned	Not mentioned	Not mentioned	Not mentioned	BMA: 2 ml
McCoy^21^	2019	Biobank (Not mentioned)	131 (47 male) compared to 1627 (588 male) with uneventful healing	Upper or lower extremity fractures	Control group: 64.3 ± 15.0; non‐union group: 66.8 ± 12.7	Not applicable
Zhang^22^	2018	May 2012–April 2015	24 (11 male) compared to 24 (11 male) with uneventful healing	Fibular head fracture	Control group: 41.5 ± 11.6; non‐union group: 40.4 ± 11.1	Not mentioned
Huang^23^	2018	2012–2016	1229 (346 non‐unions of which 199 males; 883 unions of which 505 males)	Tibial diaphysis: 113/315; femur diaphysis: 98/233; humeral shaft: 82/188; ulnar shaft: 39/117; femur neck: 14/30 (Non‐union/Union)	Non‐union: 46.1 ± 8.1; Union: 44.7 ± 8.3	Not applicable
Granchi^24^	2017	Not mentioned	26 (15 male)	Tibia: 11; femur: 11; humerus: 3; not reported: 1	39.6 ± 14	Not applicable
Sathyendra^25^	2014	2005–2010	Atrophic non‐union: 33 (14 male); normal healing: 29 (18 male)	Non‐union: femur: 13; tibia; 18; ulna: 2. Normal healing: femur: 10; tibia; 15; humerus: 4.	Atrophic non‐union: 48.6 years; normal healing: 47.3 years	Not applicable
Zeckey^27^	2011	2000–2008	50 compared to 44 patients with uneventful healing	Femur: 21; tibia: 29	37.5 ± 2.0	Not applicable
Dimitriou^28^	2011	2005–2007	62 (45 male) compared to 47 (33 male) with uneventful healing	Tibia: 41; femur: 18; humerus: 2; ulna: 1	43.9 years (range, 19–65 years)	Not applicable
Marchelli^26^	2009	Not mentioned	Atrophic non‐union: 16 (16 male); healed ‐ 6 months: 18 (18 males); healing ‐ 1 month: 14 (14 males)	Atrophic non‐unions: Tibia: 7; radius: 1; radius + ulna: 3; humerus: 2; femur: 3. Healed: Tibia: 9; radius: 2; radius + ulna: 4; humerus: 1; femur: 2. Healing: Tibia: 8; radius + ulna: 2; humerus: 2; femur: 2.	Atrophic non‐union: 28.1 ± 5.9 years; healed: 32.2 ± 5.7 years; healing: 31.4 ± 7.1 years	Not mentioned
Xiong^29^	2009	Not mentioned	Not mentioned	Not mentioned	Not mentioned	Not mentioned
Seebach^30^	2007	Not mentioned	Not mentioned	Male: 41 ± 15; female: 42 ± 13	Not mentioned	Not mentioned
Henle^31^	2005	Jan 2002–Jan 2004	15 (12 males) from non‐unions and matched group with uncomplicated unions	Tibia: 11; femur: 2; humerus: 1; forearm: 1	47 years (range, 20–75 years)	Not applicable

Abbreviations: ASIS, anterior superior iliac spine; BMA, bone marrow aspirate.

### 3.2 Studies characteristics

The study characteristics of the non‐union tissue and relevant tissue are outlined in Table 4.^8^, ^9^, ^10^, ^11^, ^12^, ^13^, ^14^, ^15^, ^16^, ^17^, ^18^, ^19^, ^20^, ^21^, ^22^, ^23^, ^24^, ^25^, ^26^, ^27^, ^28^, ^29^, ^30^, ^31^ Non‐union was defined based upon radiographic and clinical examination, with minor variations between studies. Samples of non‐union tissue and relevant tissue were mostly obtained during the surgical treatment of non‐unions.

**TABLE 4:** Study characteristics of non‐union tissue and relevant tissue

Author	Duration of non‐union (months)	Classification	Definition of non‐union	Isolation of tissue	Cells/material isolation
Cuthbert*, ^8^	Not mentioned	Atrophic	Not mentioned	Non‐union: Fibrotic tissue lying directly between the fractured bone fragments was excised and collected; induced periosteum from centre of bone defect area; and bone marrow	Colony forming unit fibroblast (CFU‐F) assay; trilineage differentiation; histological analysis of vessel number, size and area; immunohistochemistry (CD45, SDF1, VEGF, BMP‐2); flow cytometry; qPCR; matrigel‐based angiotube formation assay
Wei*, ^9^	Not mentioned	Atrophic	Not mentioned	Tissue samples were collected intra‐operatively from (i) non‐union tissues of atrophic bone; and (ii) healing callus around internal fixation plates in normal controls. Collected tissues were cut into “small” pieces	RNA isolation, miRNA microarray, bioinformatics of target genes, qPCR, Western blot, luciferase reporter assay
Burska**, ^18^	Not mentioned	Not mentioned	Failure of the fracture to progress to healing radiographically with the presence of bridging callous on at least 3 cortices by a period of 9 months	Peripheral blood	ELISA
El‐Jawhari**, ^19^	Not mentioned	Atrophic	Absence of radiological features of fracture healing (lack of callus formation in at least 3 cortices) either on plane radiographs or computed tomography scans after 9 months from fracture fixation and with ongoing pain at the NU site during ambulation	BMA; peripheral venous blood	FACS cell sorting; flow cytometry surface cytokine receptor measurement; flow cytometry—immunosuppression assay: levels of IDO, PGE2 and TGF‐β transcripts; osteogenic differentiation; RNA extraction; RT‐qPCR; proliferation (XTT colorimetric assay); ELISA
Ouyang**, ^20^	Not mentioned	Not mentioned	Not mentioned	BMA	circRNA microarray, RNA FISH, Osteogenic differentiation assay (ALP and Alizarin red staining), cck‐8 assay, RNA pull‐down assay, double luciferase reporter assay, qPCR, RNA immunoprecipitation, Western Blot
McCoy**, ^21^	Not mentioned	Not mentioned	Not mentioned	Peripheral blood	DNA was extracted from blood samples
Zhang**, ^22^	Not mentioned	Not mentioned	Not mentioned	Peripheral blood	DNA was extracted from blood samples
Wang*, ^10^	Not mentioned	Not mentioned	Not mentioned	Not applicable	Cell viability; mineralization assay; gene expression
Vallim*, ^11^	34 months (range 9–120 months)	Not mentioned	Lack of bone healing after 9 months of the fracture	Fibrous tissue interposed between the bone ends was excised, along with adjacent osseous fragments	Histology; population doubling; cell senescence; flow cytometry; osteogenic / adipogenic differentiation
Huang**, ^23^	>9 months	Not mentioned	The cessation of all healing processes and failure to achieve union within 9 months without radiographic signs of progression of the fracture callus	Peripheral blood	DNA was extracted from blood samples
Granchi**, ^24^	>3 months	Not mentioned	Not mentioned	BMA, peripheral blood	Immunoenzymatic assays
Takahara*, ^12^	14.8 months (range 4–26 months)	Pseudoarthrosis	(1) gross motion at the fracture site on physical examination; (2) bridging bone on 0 of 4 cortices on anteroposterior and lateral radiographs; (3) CT showing no purpose‐ ful cross‐sectional area of healing; and (4) evidence showing the existence of pseudocapsule and fluid collection between the fracture gap at the surgery	A small amount of pseudoarthrosis tissue (pseudocapsule) was obtained during the surgical treatment	Alizarin Red S staining, ALP activity assay, and RT‐PCR after osteogenic induction. Chondrogenic differentiation capacity was assessed via Safranin O staining and RT‐PCR after chondrogenic induction. Histological analysis and cell cultures
Schira*, ^13^	18.3 months (range, 3–100 months)	Not mentioned	Non‐unified fractures >3 months with a resorption zone wider than 1 mm (as determined by a mandatory CT scan) with no apparent potential to heal without surgical intervention	Non‐union tissue (excluding the cortex) and cancellous bone from the ipsilateral radius has been obtained at the time of operative repair	Histology, immunohistochemistry, gene expression
Han*, ^14^	11 months (range, 6–30 months)	Not mentioned	Failure of the fracture to heal 6 months or more after surgery or non‐surgical treatment	Fracture and scar tissue during surgery, which was divided into bone stump tissue, marrow cavity contents, and sticking bone scars according to the sites	Histology, immunohistochemistry, gene expression
Wang*, ^15^	Hypertrophic non‐unions: 19.88 ± 17.88 months. Atrophic non‐unions: 14.20 ± 7.42 months	Not mentioned	Failure of the fracture to heal 9 months or more after the injury	Intra‐operative biopsy samples	Immunohistochemistry
Schwabe*, ^16^	Not mentioned	Not mentioned	Time span from the initial operation until the revision surgery of a least 6 months	Intra‐operative biopsy samples for the treatment of the non‐union or removal of metalwork for the control (normal healing)	Histology, immunohistochemistry, ELISA
Sathyendra**, ^25^	Not applicable	Not applicable	Minimal callus formation 6 months after injury requiring additional surgery to achieve union	Buccal mucosal cell harvesting	SNP genotype
Ismail*, ^17^	37.2 ± 24.0 (range, 12–72)	Not mentioned	Not mentioned	Intra‐operative BM from the site adjacent to the non‐union, compared to BM from iliac crest.	Not mentioned
Marchelli**, ^26^	Atrophic non‐union: 6 to 11 months; healed: 8.5 ± 3.5 months; healing: 0.5 ± 0.5 months	Not mentioned	Not mentioned	Blood samples	ELISAs
Zeckey**, ^27^	>9 months	Aseptic tibial and femoral shaft non‐unions	Clinically and radiologically confirmed unhealed shaft fractures >9 months following the injury and osteosynthesis treatment	Peripheral venous blood sample	DNA was extracted from blood samples
Dimitriou**, ^28^	Required further intervention to achieve union	Atrophic	Cessation of all healing processes and failure to achieve union after the expected period of time, as seen clinically and radiologically	Peripheral venous blood sample	DNA was extracted from blood samples
Xiong**, ^29^	Not mentioned	Not mentioned	Fracture that does not heal 6 months after injury	Normal and non‐union callous bone samples examined	Gene expression
Seebach**, ^30^	Not mentioned	Atrophic	Not mentioned	BM cells were obtained from the iliac crest aspirate	CFU‐F; flow cytometry; osteogenic differentiation
Henle**, ^31^	>4 months	Atrophic	No bony consolidation of the fracture in conventional X‐ray films and the patient continued to report exercise induced pain 4 months after trauma + no bone healing on CT scan	Venous blood	Immunosorbent assays

Abbreviations: BM, bone marrow; BMA, bone marrow aspirate.

* Non‐union tissue.

** Relevant tissue.

### 3.3 Macroscopic characteristics of non‐union tissue

The macroscopic structure of non‐union tissue was only assessed by Han et al.’s study, whereby tough scars surrounding the site of fracture non‐union were identified.^14^ The same team also described bony sclerosis of the fracture ends and complete obliteration of the medullary canal, with fibrous connections found between the fracture fragments.^14^

### 3.4 Microscopic characteristics of non‐union tissue and relevant tissue

#### 3.4.1 Histology

Histological findings of non‐union tissue are summarized in Table 5.^8^, ^10^, ^11^, ^12^, ^13^, ^14^, ^16^ Direct comparison of histological findings between atrophic and hypertrophic union is presented in Table 6.^8^, ^11^, ^13^, ^15^, ^16^, ^32^, ^33^, ^34^, ^36^, ^45^, ^46^, ^49^

**TABLE 5:** Histological findings of non‐union tissue

Author	Classification	Histology
Cuthbert^8^	Atrophic	H&E stain of non‐union tissue: small fragments of dead bone, lack of viable osteocytes, suggesting inadequate clearance by osteoclasts. Lack of viable osteoclasts and greater percentage of pericytes, CD31^+^ and reduced number of lymphocytes compared to induced membrane tissue.
Vallim^11^	Atrophic	Connective tissue with a dense collagenous extracellular matrix, populated by fibroblast‐like cells, and areas of vascularization.
Takahara^12^	Pseudoarthrosis	Mainly fibrous tissue with variable amount of fibroblastic cells. Small vessels were sparsely populated. No ossicles or hyaline cartilage were seen in any of the sections examined.
Schira^13^	Not mentioned	Pentachrome staining revealed a heterogeneous mix of different tissues, with a domination of connective tissue and fibroblasts in non‐unions, whilst osteoid was the dominant tissue in cancellous bone. Representative TRAP staining of control cancellous bone and scaphoid non‐unions revealed enhanced osteoclasts activity in non‐unions.
Han^14^	Not mentioned	Delayed union and non‐union areas comprised a mix of different types of tissues: fracture fragments and surrounding tissues were mainly subject to fibrosis, in which the formation of new blood vessels could be seen, and a small amount of woven bone could be seen nearby. In these woven bones, Gergen Bauer's cells grew along the osteoid as cubes, suggesting active bone formations. A large number of cartilage cells existed in the intramedullary tissues, and there was no new bone and neovascularization. Bone marrow occlusion was observed, and in the fibrous tissue of adjacent bone and the gap of bone fractures, there were internal cartilage ossifications and fibrous ossifications. Scattered lamellar bone fragments were observed in some samples; these fractures were surrounded by osteoclasts, and there was a lack of osteoblasts.
Wang^10^	Not mentioned	There were no significant differences in the morphology of atrophic / hypertrophic non‐union tissues. They included MSCs, fibrocartilage cells and hyaline chondrocytes. Some sections showed very few bone islands. BMP‐2‐positive cells were present in both hypertrophic and atrophic non‐union tissue.
Schwabe^16^	Not mentioned	The tissue was a very heterogeneous mixture of fragments of lamellar bone, immature and hypertrophic cartilage, unorganized fibrous tissue and newly formed woven bone. Independent of the group, bone apposition and resorption were seen in the tissue samples. Differences between the groups were not obvious.

**TABLE 6:** Comparison of histological findings between atrophic—hypertrophic non‐unions

	Atrophic	Hypertrophic
Type of tissue		
Fibrocartilaginous tissue	33, 34	34, 46
Fibrous tissue	16, 32, 34	34, 36
Cartilaginous tissue	^16^	32, 34, 45
Collagenous extracellular matrix/connective tissue	11, 13, 32, 33	32, 33, 45
Bone tissue	No ossicles^32^; **occasional bony islands** ^15^, ^33^, ^34^ **; lack of viable osteoclasts and greater percentage of pericytes, CD31^+^ and reduced number of lymphocytes compared to induced membrane tissue** ^8^ **Mixture of lamellar and woven bone** ^16^	No ossicles^32^, ^36^; **bony islands** ^15^, ^34^, ^45^, ^46^
Necrotic bone	More prevalent^34^	‐
Bone production	Predominantly via the endochondral route^34^	Bone formation by both endochondral and intramembranous ossification^34^
Cells	‐ Generally oligocellular^32^; ‐ some areas acellular^33^ ‐ **Fibroblasts: majority of cells** 11, 13, 33 ‐ Osteoclasts: occasionally^33^ or **enhanced activity** ^13^ ‐ bipolar cells: majority of cells^33^ ‐ Cells with a stellate (possessed multiple cytoplasmic processes) or dendritic appearance^33^ ‐ Include MSCs, **fibrocartilage cells and hyaline chondrocytes** ^15^	‐ More cellular^32^ ‐ Fibroblast‐like^36^ ‐ **Include MSCs, fibrocartilage cells and hyaline chondrocytes** ^15^
Vascularization	Well vascularized^33^, ^34^, ^49^; **few vessels** 11, 32	Well vascularized^34^

Note

As only reporting on studies published after our original review^1^ would provide an incomplete picture of the differences between atrophic and hypertrophic non‐unions, we include all relevant data regardless of publication date.

References highlighted **bold**: new references published after our original review.^1^

#### 3.4.2 Immunohistochemistry

The immunohistochemical findings of non‐union tissue and relevant tissue are summarized in Table 7.^8^, ^13^, ^14^, ^15^, ^16^, ^18^, ^19^ BMPs were present in non‐union tissue.^8^, ^14^ Interestingly, Han et al. found BMP to be locally generated by non‐union tissue.^14^ Additionally, BMP antagonists were also found to be present in both normal and non‐union tissue alike.^16^ ALP and SMAD2/3 were both found to be increased in scaphoid non‐union tissue.^13^ Cuthbert et al. also confirmed the presence of SDF‐1 and VEGF in non‐union tissue.^8^

**TABLE 7:** Immunohistochemistry findings

Author	Classification	Immunohistochemistry
Cuthbert^8^	Atrophic	Presence of SDF‐1, VEGF and BMP‐2 in NU tissue. CD 45 staining: greater in induced membrane than in non‐union. Non‐union tissue contains significantly greater percentage of cells expressing (i) pericyte (13.8% vs. 4.9%), (ii) CD31^+^ endothelial cells (18.2% vs. 5.5%) phenotypic markers. Non‐union tissue had significantly reduced numbers of lymphocytes (6.8% vs. 22.2%)
Burska^18^	Not mentioned	PIGF was higher in non‐union patients, reaching significance at Days 1 and 3 (*p *< 0.05); but less marked at Day 5 (*p* = 0.09). PIGF displayed initial massive surge followed by rapid decline in non‐union patients. TGF‐beta 2 appeared higher in union group (not statistically significant). Levels of MCP‐1 and IL8 showed no clear difference between non‐union and union groups.
El‐Jawhari^19^	Atrophic	IFN‐γ, TNF‐α and IL‐1 levels similar between non‐union, union and control arms. However, lower levels of IL‐17 detected at later stages of fracture healing (vs. union and control arms)
Schira^13^	Atrophic	ALP reached higher levels in scaphoid non‐unions as opposed to cancellous bone. Likewise, immunofluorescence for phosphorylated SMAD2/3 revealed increased activity in scaphoid non‐unions.
Han^14^	Not mentioned	The depth of BMP‐2 staining in the cytoplasm increased with increasing proximity to the new bone formation region, and there was some staining of the Golgi apparatus, showing that BMP‐2 was locally generated. A wide variety of cells, including epithelial cells, smooth muscle cells around the small blood vessels, fusiform fibroblast‐like cells and chondrocyte cells, showed positive staining in the fibrous tissues, indicating osteogenesis. There was no difference in the immunostaining of fibrous tissue between the samples with and without new bone. There was no positive BMP staining in the extracellular matrix or the fibrous tissue space. Sub‐parts of view, fracture fragments were mainly fibrotic tissues and BMP‐2 staining was negative. In the surrounding tissues, especially in the sticking scars and posted plate scars, neovascular and woven bone filled in a lot of the fibrous tissues, and in the vicinity, there were stained cells, indicating BMP‐2 expression. There was a small amount of cartilage with positive staining in the cytoplasm, without expression in fibrous tissues of the closed medullary cavity. DCN expression was extensive in the interstitial fracture fragments. There was no positive staining of cartilage cells in the medullary cavity. DCN expression in the sticking scars was close to perivascular. The rate of expression of BMP‐2 was highest in the posted bone scar group, and was low in the bone ends and canal content group (*p* < 0.05). There was no significant difference between the other two groups. The fracture fragment group had the highest DCN expression, with significant differences from the other two groups; the least significant difference analysis showed that between the fracture fragment group and the other two groups, *p* < 0.05; between the other two groups, *p* > 0.05
Wang^15^	Atrophic/hypertrophic	The mean optical density of BMP‐2 was 0.154 ± 0.041 in hypertrophic non‐union tissue, 0.137 ± 0.037 in atrophic non‐union tissue, there was no significant difference between the 2 groups (*p *> 0.05). The mean optical density of BMP‐2 was 0.148 ± 0.040 in the 20‐ to 35‐year‐old group, 0.142 ± 0.040 in the 35‐ to 50‐year‐old group, 0.146 ± 0.056 in the more than 50‐year‐old group, there was no significant difference among the three groups (*p *> 0.05). The mean optical density of BMP‐2 was 0.145 ± 0.037 in the 9–12 months group, 0.147 ± 0.0400 in the 13–24 months group, 0.145 ± 0.054 in the more than 24 months group, there was no significant difference among the 3 groups (*p *> 0.05).
Schwabe^16^	Atrophic	Bone morphogenic antagonists were demonstrated in non‐union and control tissue.

In terms of relevant tissue, peripheral PIGF levels were found to be higher in non‐union patients, with an initial surge followed by a rapid decline. Both TGF‐ß2^20^ and IL‐17^19^ on the contrary were reported to be lower in non‐union patients.

#### 3.4.3 Analysis of vessel calibre, area and density

Blood vessels were present in cases of hypertrophic non‐unions, with a varying density (Table 8).^8^, ^13^, ^16^ Only one study assessed vessel density in atrophic non‐unions, reporting a 2.4‐fold increase when compared against that of induced periosteal membrane (control group).^8^ However, both vessel calibre and median area were smaller in non‐union tissue in this study.^8^ All these reaffirms histological findings whereby vascular tissue was found to be present in both atrophic and hypertrophic non‐unions.^11^, ^12^, ^14^, ^16^

**TABLE 8:** Analysis of vessel density

Author	Analysis of vessel density
Cuthbert^8^	2.4‐fold increase in non‐union tissue when compared against induced membrane tissue. Both calibre and median internal vessel area of bloods vessels in NU tissue were smaller compared to induced membrane.
Schira^13^	Angiogenesis in scaphoid non‐unions is similar to cancellous bone. Blood vessels and endothelial cells were detected by immunohistochemical staining of PECAM‐1 in non‐unions and controls revealing similar levels of angiogenesis in both tissues.
Schwabe^16^	Histology: Vessels were present in all investigated samples without a difference between the tissue from non‐union and control patients. Immunohistochemistry: well vascularized but also unvascularized areas with no difference between the non‐union and the control tissue.

### 3.5 Cellular characteristics and functions

#### 3.5.1 Cell surface protein expression

Altogether, four studies evaluated the expression of cell surface protein using flow cytometry (Table 9).^11^, ^12^, ^17^, ^19^ Non‐union tissue was found to be positive for MSC‐related markers CD73,^11^, ^17^ CD90^11^, ^17^ and CD105,^11^, ^12^, ^17^ but negative for haematopoietic markers CD14,^17^ CD34,^17^ CD45^12^, ^17^ and HLA‐DR.^17^ El‐Jawhari et al. demonstrated in relevant tissue in the form of BM‐MSC harvested from the iliac crest of non‐union patients to express lower levels of IL‐1R1 compared to controls.^19^

**TABLE 9:** Cell surface protein expression

Author	Cell surface protein expression (flow Cytometry)
El‐Jawhari^19^	Uncultured non‐union CD271 high CD45low cells expressed fewer transcripts of IL‐1R1 compared to union cells. No significant difference in other cytokine receptor transcripts (CD119, CD120a and CD217). IL‐1R1 surface protein less in uncultured non‐union CD271high CD45low cells (*p *= 0.049).
Vallim^11^	Compared to BM MSC and osteoblasts, non‐union MSCS: Homogeneously expressed CD90 and CD73. The percentage of cells expressing CD105 was significantly lower in comparison with BM MSCs, and similar to that of osteoblasts. CD146^+^ positive cells was lower compared to BM MSCs. When evaluating the percentage of cells simultaneously expressing both markers, NUSC had 3.78% ± 4.0% of CD105^+^/CD146^+^ cells, whilst osteoblasts and BMSC had 0.77% ± 0.9% and 39.6% ± 25.7% respectively. Collectively, these results confirmed that NUSC indeed contained cells of the osteoblastic lineage, whose surface marker profile resembles that of cells in late‐stage differentiation.
Takahara^12^	Consistently positive for MSC‐related markers such as CD29, CD44, CD105 and CD166. The cells were negative for haematopoietic‐lineage markers such as CD31, CD34, CD45 and CD133.
Ismail^17^	There was positive expression of CD105, CD73 and CD90 for at least 95%, negative expression of CD45, CD34, CD14 or CD11b, CD79a or CD19, and HLA‐DR.

Abbreviations: BMSC, bone marrow stromal cells; MSC, mesenchymal stem cells; NUSC, non‐union stromal cells.

### 3.6 Morphology, viability, proliferation and cellular senescence

The (i) cell morphology, viability and proliferation of non‐union tissue; and (ii) the effect of non‐union serum on proliferation of BM‐MSCs are outlined in Table 10.^8^, ^10^, ^11^, ^12^, ^17^, ^19^ Overall, non‐union MSCs were found to have comparable proliferative capacities and viability to that of BM‐MSCs.^8^, ^10^, ^11^, ^12^, ^17^ On the contrary, non‐union serum was found to have a negative effect on MSC proliferation.^19^ Comparing the cell senescence rates of non‐union MSCs and those of bone marrow MSCs, Vallim et al. found no difference between the two groups.^11^

**TABLE 10:** Cell culture characteristics and functions

Author	Classification	Intervention	Cell morphology	Cell viability (MTT‐Test)	Cell proliferation
Cuthbert^8^	Atrophic	Not applicable	Not applicable	Not applicable	Cells isolated from non‐union tissue behave similarly to that of BMA, readily forming colonies. CFU‐F from non‐union tissue were comparable to that of induced membrane tissue, indicating no difference in MSC content between the two tissues.
El‐Jawhari^19^	Atrophic	MSC cultured in non‐union serum vs. union serum	Not applicable	Not applicable	Non‐union serum has negative effect on MSC proliferation (*p *= 0.031).
Wang^10^	Not mentioned	Chordin, Noggin and Gremlin expression knockdown	Not applicable	The cell viability of MSCs remained unchanged with PSI. By contrast, the cell viability of PEI25 kDa‐treated MSCs dramatically dropped to 20% of the original value when the polymer concentration reached 15 μg/ml.	Not applicable
Vallim^11^	Not mentioned	Non‐union MSCs, BM MSCs and osteoblasts were transplanted into the subcutaneous tissue of immunodeficient mice	Not applicable	Not applicable	Non‐union MSCs had proliferative and rates comparable to BM MSCs and osteoblasts. The percentage of cells staining positive for b‐galactosidase activity in non‐union MSCs cultures was comparable to those observed in BM MSCs and osteoblasts.
Takahara^12^	Pseudoarthrosis	Not applicable	Fibroblast‐like spindle shape	Not applicable	Could be cultured through at least 10 passages, with minimal decline in their proliferative capacity
Ismail^17^	Not mentioned	Not applicable	Not applicable	Non‐union: viability of 87.1% (81.7%–90.8%); iliac crest: 89.8% (84.7%–94.5%). No differences were found between the two sources of MSCs (*p* = 0.175).	Not applicable

Abbreviations: BMA, bone marrow aspirate; BMP, bone morphogenic protein.

#### 3.6.1 Mineralization and Alkaline phosphatase (ALP) activity assay

The outcomes of mineralization assay for non‐union tissue are outlined in Table 11.^10^, ^11^, ^12^, ^13^, ^24^, ^26^ The findings of the four studies which evaluated ALP activity and its mRNA expression are outlined in Table 12.^12^, ^13^, ^24^, ^26^

**TABLE 11:** Osteocalcin expression and mineralization assay

Author	Classification	Intervention	Osteocalcin	Mineralization Assay
Wang*, ^10^	Not mentioned	Chordin, Noggin and Gremlin expression knockdown	Promoted by Chordin knockdown, more strongly than Gremlin. Decreased by Noggin knockdown	The osteogenic differentiation of MSCs isolated from non‐unions was lower than those isolated for patients with uncomplicated healing
Vallim*, ^11^	Not mentioned	Non‐union MSCs, BM MSCs and osteoblasts were transplanted into the subcutaneous tissue of immunodeficient mice	Not applicable	Non‐union MSCs deposited mineralized matrix positive for Von Kossa, similarly as BM MSCs and osteoblasts
Granchi**, ^24^	Not mentioned	Regenerative approach consisted in a minimally invasive administration of autologous bone marrow cells expanded in good manufacturing practice (GMP) facilities	After regenerative treatment: At the time of BM harvesting, intact osteocalcin and N‐terminal/midregion osteocalcin levels were comparable to the reference values of healthy individuals. N‐terminal/midregion osteocalcin decreased after 6 weeks. At 24 weeks, concentrations were similar to those observed before treatment. Intact osteocalcin and N‐terminal/midregion osteocalcin levels were significantly decreased at 6 weeks in patients healed after 24 weeks, to increase afterwards, with changes not significantly different from baseline values.	Not applicable
Takahara*, ^12^	Pseudoarthrosis	Not applicable	Its expression under osteogenic conditions was upregulated compared with those under control conditions, and had a similar pattern to that shown by BMSCs.	Formed a mineralized matrix as observed on Alizarin Red S staining, contrasting with the absence of a mineralized matrix under control conditions after the same duration
Schira*, ^13^	Not mentioned	Not applicable	Similar expression pattern in non‐union tissue and controls.	Not applicable
Marchelli**, ^26^	Not mentioned	Not applicable	Serum osteocalcin levels in non‐unions were similar to healed and healing fractures (*p *> 0.05)	Not applicable

* Non‐union tissue.

** Relevant tissue.

**TABLE 12:** ALP activity and ALP related mRNA expression

Author	Classification	Intervention	ALP activity assay	ALP mRNA
Granchi**, ^24^	Not mentioned	Regenerative approach consisted in a minimally invasive administration of autologous bone marrow cells expanded in good manufacturing practice (GMP) facilities	After regenerative treatment: At the time of BM harvesting, levels generally tended to be higher than reference values of healthy individuals. After 6 and 12 weeks from surgery, a significant increase was observed. At 24 weeks, concentrations were similar to those observed before treatment. Bone‐specific ALP correlated to the imaging results collected at 12 and 24 weeks. Its variation along the healing course differed in patients who had an early consolidation (at 12 weeks). A remarkable decrease in ALP was observed at all time points in a single patient who experienced a treatment failure.	Not applicable
Takahara*, ^12^	Pseudoarthrosis	Not applicable	ALP activity increased with time and declined on Day 28. By contrast, under control conditions, ALP activity in culture remained low between days 7 and 28. ALP activity under osteogenic conditions was significantly higher than that under control conditions on days 14 and 21 (*p* = 0.0179 and 0.0489 respectively).	Its expression under osteogenic conditions was upregulated compared with those under control conditions, and had a similar pattern to that shown by BMSCs.
Schira*, ^13^	Not mentioned	Not applicable	Not applicable	ALP was significantly upregulated across all non‐unions.
Marchelli**, ^26^	Not mentioned	Not applicable	Serum ALP levels in non‐unions were similar to healed and healing fractures (*p *> 0.05)	Not applicable

Abbreviations: BMP, bone morphogenic protein; ALP, alkaline phosphatase; mRNA, messenger RNA; CFU, colony forming units

* Non‐union tissue.

** Relevant tissue.

### 3.7 Molecular characteristics

#### 3.7.1 Protein and micro RNA levels

Wang et al. utilized Western blot assay to evaluate the expression of p‐SMAD1/5/8 protein in non‐union tissue and that of ‘normal’ fracture healing.^10^ The same team also reported decreased expression of p‐SMAD1/5/8 in MSCs isolated from patients with non‐union.^10^ Interestingly, chordin knockdown was found to rescue the osteogenic capacity of MSCs of non‐union patients.^10^ Wei et al. identified the four micro RNAs (miRNAs) significantly upregulated in atrophic non‐unions (hsa‐miR‐149∗, hsa‐miR‐221, has‐miR‐628‐3p and hsa‐miR‐654‐5p); and upon transfection of BM‐MSCs with the same four miRNAS, significantly decreased its expression of ALPL, PDGFA and BMP2.^9^ Marchelli et al. found that serum osteocalcin levels in non‐unions were similar to healed and healing fractures (*p *> 0.05).^26^ Interestingly, Granchi et al. demonstrated that osteocalcin and N‐terminal/midregion osteocalcin levels to be significantly decreased at 6 weeks, followed by a return to levels similar to baseline values.^24^

#### 3.7.2 Gene expression and genetic predisposition

Several authors have examined the expression of different genes in the non‐union tissue^8^, ^10^, ^12^, ^13^, ^14^ and relevant tissue.^19^, ^21^, ^22^, ^23^, ^25^, ^27^, ^28^, ^29^ Summaries of their results are outlined in Tables 12, ^12^, ^13^, ^24^, ^26^ and 13.^8^, ^10^, ^12^, ^13^, ^14^, ^19^, ^21^, ^22^, ^23^, ^25^, ^27^, ^28^, ^29^

**TABLE 13:** Gene expression/genetic predisposition

Author	Gene expression/genetic predisposition
Non‐union tissue	
Cuthbert^8^	Genes with endothelial regulatory role: FLT1 and ANGPTL4 were significantly lower in NU tissue compared with BMMSC and IP MSCs. MCAM1 and PTN: increased in NU tissue, with PTN reaching statistical significance. Wnt pathway genes: FZD4 & WNT2: decreased in NU MSCs; no difference with DKK1, DKK2, SOST, KREMEN1 SOX9 & BMP2: increased in NU tissue when compared against IP tissue, with only SOX 9 being statistically significant.
Wang^10^	Chordin, Noggin and Gremlin: higher in bone non‐union isolated MSCs, whilst the expression of BMP‐7 was lower. ID1 and ID3: downregulated in non‐union MSCs. Chordin knockdown is an ideal target for enhancing the osteogenic differentiation of MSCs in patients with bone non‐union. Chordin knockdown rescued the osteogenic capacity of MSCs isolated from patients with bone non‐union.
Takahara^12^	RUNX2 under osteogenic conditions: upregulated compared with those under control conditions, and had a similar pattern to that shown by BMSCs. The mRNA of aggrecan, Col II, Col X, SOX5, and SOX9 after a 21‐day chondrogenic induction was not expressed. Glycosaminoglycan was extensively present in sections from BMSC pellets, and a high expression of those chondrocyte‐related genes was observed in BMSC pellets after a 21‐day chondrogenic induction.
Schira^13^	Noggin: significantly downregulated in non‐union tissue. BMP‐7 and pro‐osteogenic FGFs, FGF‐9 and FGF‐18: undetectable in both non‐unions and control cancellous bone. FGF‐2: not differentially expressed Cyclin D1: significantly upregulated in non‐unions. WNT3A: not detectable in both tissues, whilst WNT5A was upregulated in non‐unions. MMP‐9 & MMP‐13: significantly upregulated in non‐unions. PECAM‐1: similar expression levels in non‐unions and controls. RUNX2: hardly detectable in non‐unions and controls. Significant upregulation of RANKL in non‐unions (20‐fold), OPG and NFATc1, regardless of duration of the non‐union. The RANKL receptor RANK (receptor activator of nuclear factor κB) and M‐CSF: slightly but not significantly upregulated. ATF4 (Activating Transcription Factor 4): unchanged.
Han^14^	BMP‐2: expressed in non‐union tissue; this was highest in the posted bone scar and lowest in the bone ends. The expression in the posted bone scar was significantly different to the canal content and bone ends groups (bone ends < marrow cavity < posted bone scar). Decorin: was expressed in three different parts of the non‐union area, and was highest in the bone ends. The expression level in the bone ends group was significantly different to the canal content and posted bone scar groups (*p* < 0.05).
Relevant tissue	
El‐Jawhari^19^	Osteogenic markers: Significantly lower levels of *ALPL*, *BGLAP*, *SPARC* and *SPP1* in uncultured non‐union BM cells. NU BM‐MSCs cultured in non‐union serum had less ALPL transcripts when compared to NU BM‐MSCs cultured in union serum OR union BM‐MSCS cultured in both union/ non‐union serum. *BGLAP*, *SPP1* and *SPARC*: comparable in both serum cultures. Markers of immunosuppression (in uncultured or minimally cultured MSC): *TGF*‐*β1 and PTGES2* similar between NU and U BM‐MSC. *BST2*: lower in NU BM‐MSC. *S100A8 (immunoregulatory molecule)*: higher levels detected in NU BM‐MSC. *BST2* transcript levels were positively correlated with *ALPL*, *BGLAP*, *SPARC*, *EGFR*, *FGFR1 & FGFR2*; suggesting *BST2* link to osteogenic and proliferation of BMMSC. Cytokine treated NU BM‐MSCs: lower *IDO*, *TGF*‐*β1 and PTGES2* than union BM‐MSCs in matched serum culture. Union BM‐MSCs express few transcripts of *IDO*,*TGF*‐*β1 and PTGES2* when treated in NU serum cultures. Markers of immunosuppression (in culture‐expanded MSC): *IDO* levels were similar whether treated by IFN‐γ alone or combined with *TNF*‐*α*, *IL*‐*1 or IL*‐*17*. *IDO* levels were similar between NU and U BM‐MSCS. *LAP* (surface *TGF*‐*β1*) were similarly increased in NU and U BM‐MSCS after cytokine treatment. Comparable immunosuppressive functions of culture‐expanded NU‐ and U‐MSCs.
McCoy^21^	The most strongly associated SNP is located in Calcyon (CALY). Among the loci associated with non‐union (*p* < 5e–7), one notable region spans the tachykinin receptor‐1 (TACR1) gene, also referred to as the neurokinin or substance P receptor.
Zhang^22^	CtBP2, but not CtBP1 (only slightly increased), is significantly upregulated in atrophic non‐union tissue compared to healthy controls. Osteoblast isolated from non‐union tissue also had the same upregulation compared to healthy controls. SPHK1, Dkk‐1 and CDH2:significantly upregulated in all atrophic non‐union tissues p300, RUNX2 and BMP2: downregulated in all atrophic non‐union tissues CtBP2 forms a transcriptional complex with p300 and RUNX2. More specifically, CtBP2 plays an inhibitory role in regulating p300‐RUNX2 complex formation. The CtBP2‐p300‐RUNX2 transcriptional complex inhibits the expression of genes involved in bone formation and differentiation. An elevated NADH level upregulates RUNX2 target gene levels in osteoblasts.
Huang^23^	SNP rs2297514: significant association with the fracture healing process after adjusting for age and gender (OR = 1.38, *p* = 0.0005). The T allele of rs2297514 significantly increased the risk of a non‐union during the fracture healing process by 38% compared to the C allele. Significance could only be observed in the tibial diaphysis subgroup (not for femur/humerus/ulna).
Sathyendra^25^	Five SNPs on four genes were significant, with three having an OR > 1, indicating that the presence of the allele increased the risk of non‐union. rs2853550 SNP had the largest effect (OR = 5.9, *p* = 0.034), was on the IL1B gene, which codes for IL1 beta. rs2297514 SNP (OR = 3.98, *p* = 0.015) & rs2248814 SNP (OR = 2.27, *p* = 0.038): on the NOS2 gene coding for nitric oxide synthase. Two SNPs had an OR of <1, indicating that the presence of the allele may be protective against non‐union: rs3819089 SNP (OR = 0.26, *p* = 0.026) was on the MMP13 gene for MMP13, and the rs270393 SNP (OR = 0.30, *p* = 0.015) was on the BMP6 gene for BMP6.
Zeckey^27^	PDGF haplotype: significantly associated with long bone non‐unions of the lower limb following fracture. No major influence of single polymorphisms only within the genes encoding for the other observed mediators involved in fracture healing. MMP‐13 polymorhipsm: trend towards association with uneventful healing
Dimitriou^28^	Two specific genotypes (G/G genotype of the rs1372857 SNP, located on NOGGIN and T/T genotype of the rs2053423 SNP, located on SMAD6) are associated with a greater risk of fracture non‐union.
Xiong^29^	ADAMTS18 level: significantly lower in subjects with non‐union fractures as compared to subjects with normal‐healing fractures. Decreased in vivo ADAMTS18 expression might thus potentially contribute to the non‐healing of skeletal fractures. TGFBR3 level: is significantly lower in normal skeletal fracture subjects as compared to non‐union skeletal fracture subjects.

Takahara et al. discovered that non‐union tissues behaved in a similar fashion to that of BM‐MSCS, whereby osterix and bone sialoprotein expression were both upregulated in non‐union tissue cultured under osteogenic conditions, when compared against control conditions.^12^ Even more interestingly, under osteogenic conditions, Takahara et al. found that the expression of bone sialoprotein had a similar pattern to that shown by BM‐MSCs.^12^ Schira et al. reported similar patterns of *Dickkopf*‐*1* expression in both scaphoid non‐union tissue and controls (cancellous bone adjacent to non‐union site).^13^ In terms of osteocalcin expression of non‐union MSCs, both Takahara and Schira et al. found this to be similar to that of BM‐MSCs (control).^12^, ^13^

Studies on relevant tissue have also investigated genetic predisposition to fracture non‐union and identified numerous polymorphisms and genotypes associated with the increased risk of developing non‐union (Table 13).^21^, ^22^, ^23^, ^25^, ^27^, ^28^, ^29^

#### 3.7.3 Comparison between atrophic and hypertrophic non‐unions

Table 14, ^8^, ^13^, ^15^, ^16^, ^19^, ^32^, ^33^, ^34^, ^35^, ^36^, ^37^, ^38^, ^39^ provides a summarized comparison between tissues (non‐union tissue and relevant tissue) obtained from patients with atrophic and hypertrophic non‐unions.

**TABLE 14:** Comparison between atrophic/hypertrophic non‐union tissue

Type of analysis	Atrophic	Hypertrophic
Histology	**Table ** **6**	
Immunohistochemistry	**SMAD2/3 revealed increased activity in non‐unions** ^13^ **Close vicinity to immature osteoid trabeculae** ^35^ **SDF‐1, VEGF, BMP‐2 present in non‐unions** ^8^ **IL‐17 levels lower at later stages of fracture healing in non‐union BM‐MSC. IFN‐γ, TNF‐α, and IL‐1 levels in non‐union group similar to union and control group** ^19^	**‐**
Vessel density	No difference in the median vessel count between atrophic/hypertrophic non‐unions^34^ **2.4‐fold increase in non‐union tissue when compared against induced membrane tissue** ^8^	No difference in the median vessel count between atrophic/hypertrophic non‐unions^34^
Cell surface antigen profile	Less than 1% of NUSC and BMSC were positive for CD34 and CD45, whilst 78% ± 14% of NUSC and 92% ± 7% of BMSC were positive for CD105^33^ **Lesser IL‐1R1 surface protein and transcripts in uncultured non‐union BMMSC; whilst no significant difference in IFNGR1, TNFRS1A AND IL‐17RA when compared to union group** ^19^	Positive for MSC‐related markers CD13, CD29, CD44, CD90, CD105, and CD166, but negative for hematopoietic markers CD14, CD34, CD45, and CD133^36^
Cell morphology	Cells formed a uniform monolayer of elongated cells that had few cellular extensions^32^	Also consisted of elongated cells, but the cells were more cuboidal, having cellular extensions in a multilayer^32^
Cell Proliferation	Cells differentiate along each mesenchymal lineage^33^ **Cells isolated from non‐union tissue behave similarly to that of BMA, readily forming colonies** ^8^	Significantly inferior to that of fracture haematoma cells^36^
ALP Activity	No differences between atrophic/hypertrophic non‐unions^32^ **Higher levels in scaphoid non‐unions as opposed to cancellous bone** ^13^ Markedly lower than that for BMSC cultures^33^	No differences between atrophic/hypertrophic non‐unions^32^ No difference with controls^37^
Osteocalcin	Very low levels^32^	Very low levels^32^; higher than in human dermal fibroblasts^36^ The expression of osteocalcin under osteogenic conditions was higher than under undifferentiated conditions in the control group^36^
BMPs	**No significant difference in BMP‐2 levels between atrophic/hypertrophic non‐unions** ^15^ **BMPs antagonists present in non‐union tissue and controls** ^16^	**No significant difference in BMP‐2 levels between atrophic/hypertrophic non‐unions** ^15^ BMP‐2: present in the fibrous tissue of the non‐union^39^ BMP‐7: absent^39^
MMP	‐	MMP‐7 and MMP‐12 were present^38^
Mineralization Assay	Significant reduction in the MSCs capacity to differentiate along an osteoblastic lineage compared to BMSC^33^	Higher than haematoma cells^36^ Very low mineralization potential and significantly lower than ‘normal’ human osteoblasts^37^ Under osteogenic conditions, mineralization was significantly higher than that of fracture haematoma cells, in contrast to undifferentiated conditions^36^

Note

As only reporting on studies published after our original review^1^ would provide an incomplete picture of the differences between atrophic and hypertrophic non‐unions, we include all relevant data regardless of publication date.

References highlighted **bold**: new references published after our original review.^1^

#### 3.7.4 Effect of interventions on non‐union tissue and relevant tissue

Table 15, ^10^, ^19^ outlines the effects of interventions on the non‐union tissue,^10^ and BM‐MSC cultured in serum taken from non‐union patients (relevant tissue).^19^

**TABLE 15:** Effect of interventions

Author	Wang^10^	El‐Jawhari^19^
Type of Intervention	Chordin, Noggin and Gremlin expression knockdown	BM‐MSC cultured in: ‐ Non‐union and union serum (proliferation assay) ‐ Cytokine‐treatment (IFN‐γ, TNF‐α, IL‐1 and IL‐17)
Cell Proliferation	Not applicable	Non‐union serum has negative effect on BM‐MSC proliferation (*p *= 0.031).
Transforming Growth Factor‐β1	Not applicable	Lower levels in cytokine treated (IFN‐γ, TNF‐α, IL‐1 and IL‐17) NU BM‐MSC
Osterix	Promoted by Chordin knockdown, more strongly than Gremlin. Decreased by Noggin knockdown	Not applicable
Osteocalcin	Promoted by Chordin knockdown, more strongly than Gremlin. Decreased by Noggin knockdown	Not applicable
Mineralization Assay	Chordin knockdown rescued the osteogenic ability of hBMSCs isolated from patients with non‐union	Not applicable
Col1a1	Promoted by Chordin knockdown, more strongly than Gremlin. Decreased by Noggin knockdown	Not applicable

## 4 DISCUSSION

Fracture non‐union represents a significant public health problem with detrimental socioeconomic costs. In addition to productivity losses, the direct treatment cost of established non‐union in the UK has been estimated to be in the regions of £7,000 and £79,000 per person, dependent on its complexity.^40^ With multiple pathophysiological factors influencing its progression, fracture non‐union remains a challenging condition to treat.^41^ The improved understanding of its pathophysiology has seen the evolution with the treatment of non‐unions, from prolonged immobilization in the 1950s^42^ to the modern techniques of biological stimulation and polytherapy.^43^

The commonest macroscopic appearance of non‐unions is soft tissue interposition between fracture fragments.^14^, ^42^, ^44^ Han et al.’s study furthered this description, reporting bony sclerosis of fracture ends and complete obliteration of medullary canal.^14^ Additionally, non‐union tissue colour and its surrounding fluid are also important characteristics used to differentiate between septic and aseptic non‐unions (white tissue and clear surrounding fluid: aseptic; yellowish tissue and murky surrounding fluid: septic). Taken altogether, macroscopic appearances of the fracture site immediately visible to the treating surgeon in the operating theatre could serve as a powerful visual marker, aiding the confirmation/suspicion of a septic non‐union. More importantly, it could support surgeons with prompt surgical decision and the swift treatment of septic non‐unions.^1^

In terms of histological analysis, several similarities exist between atrophic and hypertrophic non‐unions. Firstly, fibrous, cartilaginous and connective tissues were historically reported to be the tissue types common to both atrophic and hypertrophic non‐unions.^32^, ^33^, ^34^, ^36^, ^45^, ^46^ Studies included in this systematic review^11^, ^13^, ^16^ confirm these findings. Secondly, bony islands were not always present in both atrophic^15^, ^32^, ^33^, ^34^ and hypertrophic non‐unions.^15^, ^32^, ^34^, ^36^, ^45^, ^46^ Thirdly, whilst fibroblast‐like cells account for the majority of the population in both atrophic and hypertrophic non‐unions,^11^, ^13^, ^33^, ^36^ MSCs were still present in both tissues.^15^ However, several differences also exist. Atrophic non‐unions contain a mixture of lamellar and woven bone,^16^ with a prevalence of necrotic bone,^8^, ^34^ lack of viable osteocytes and osteoclasts,^8^ and a predominance of endochondral bone formation.^34^ In contrast, bone formation in hypertrophic non‐unions were reported to occur equally through both endochondral and intramembranous ossification.^34^ Furthermore, cellular density was lower in atrophic non‐unions, with some areas being completely acellular.^32^, ^33^ Collectively speaking, these differences in both the cellularity and local environment may account for the higher failure rate observed following revision surgery in atrophic non‐union cases.^47^

Contrary to common historical belief that atrophic non‐unions are relatively avascular and inert,^34^, ^48^ several authors have confirmed the presence of vascular tissue, evidenced by histological analysis of atrophic^11^, ^32^, ^33^, ^34^, ^49^ and hypertrophic^34^ non‐union tissues, with no major differences between the two.^34^ Similar to the study by Reed et al.,^34^ vessel density of non‐union tissue in new studies was largely found to be at similar levels in non‐unions and cancellous^13^ or healing bone.^16^ Interestingly, Cuthbert et al. reported a 2.4‐fold increase in the vessel density of atrophic non‐union tissue, although the calibre and median internal vessel area were found to be smaller when compared against controls.^8^ These findings are promising as it highlights a research area which has the potential to restore and enrich local angiogenesis, and ultimately successful fracture healing.

Bajada et al. first reported in 2009 the presence of cells positive for MSCs‐related markers and negative for haematopoetic markers in non‐union tissue.^33^ This was later confirmed by other authors, whereby non‐union tissue was found to contain biologically active cells with the potential to differentiate into osteoblastic, chondrogenic and adipogenic lineages.^11^, ^12^, ^17^, ^36^, ^50^

With regard to culture characteristics of the non‐union tissue, only a few of the current list of studies assessed cell morphology, viability and proliferation. Both studies by Cuthbert et al. and Vallim et al. found the proliferative capacity of MSCs isolated from non‐union tissue to be comparable to that of BM‐MSCs.^8^, ^11^ Furthermore, the proliferative capacity of non‐union MSCs was found to have minimal decline following multiple passages.^12^ However, when compared against studies published in our previous review,^1^ we found an inconsistency in the reported findings on culture characteristics. This could be explained by the variability in the type of non‐union tissue examined, the geographical location of non‐union tissue and sample size.

Cell senescence have been found to impair the regenerative and therefore healing potential of MSCs and differentiated cells in non‐union tissue.^51^ There is, however, variation in terms of rates of senescence of non‐union tissues found in the literature—Vallim et al. reported senescence rate to be no different,^11^ whereas Bajada et al. reported increased proportion of senescent non‐union MSC when compared against BM‐MSC.^33^ Further work is therefore warranted since the influence of contributory factors (such as repeated cellular replication and stress) and pathways leading to the genomic damage in senescent non‐union MSCs remains unknown.

Bone morphogenic protein (BMP) plays a key role as a signalling molecule in promoting the MSC osteoblastic and chondrogenic differentiation and has therefore been extensively studied given its important role in the field of bone regeneration.^52^, ^53^ Interestingly, studies have reported evidence of BMP signalling and generation in non‐union MSCs,^8^, ^14^, ^49^ with no difference in BMP expression between atrophic and hypertrophic non‐unions.^15^ Noteworthy, BMP expression was found to be low in the bone ends and canal contents of the non‐union site, and absent in the extracellular matrix.^14^ The effects of BMP on non‐union cell cultures in vitro have also been assessed, with improved osteogenic differentiation and increased ALP levels of osteocalcin expression and mineralization potential observed following addition of BMP.^54^, ^55^

Studies by Wang et al. and Fajardo et al. have further shed light on the important topic of homeostasis between gene expression of BMP and its inhibitors (Chordin, Noggin and Gremlin).^10^, ^39^ Both studies identified reduced BMP‐7 gene expression and elevated levels of Chordin, Gremlin and Noggin.^10^, ^39^ Wang et al. went on to investigate the effects of Chordin, Gremlin and Noggin knockdown—reporting increased expression of osterix, osteocalcin and collagen following Chordin and Gremlin knockdown.^10^ Furthermore, they also demonstrated Chordin knockdown to rescue the osteogenic ability of non‐union cells.^10^ Taken altogether, these findings support the idea of imbalance expression between BMP and its inhibitors driving the pathophysiology of impaired bone healing observed in non‐union MSCs.^16^, ^39^, ^56^

Matrix metalloproteinases (MMP) are important key player, which modulate bone remodelling and repair. Disruption to either MMP or their inhibitors could result in disorders of fracture healing.^38^ In vitro studies on hypertrophic non‐union tissues have found MMP to bind directly and degrade BMP‐2, known to be an osteoinductive molecule.^38^ Furthermore, non‐union tissues were found to have an upregulation of MMP‐7, MMP‐9 and MMP‐17 genes.^13^, ^38^ All these findings highlight the potential role of MMP as one of the key players in the pathogenesis of fracture non‐union.

Although Dkk‐1 is well known as an antagonist of the Wnt signalling pathway inhibiting osteogenic differentiation,^33^, ^57^ Dkk‐1 expression by non‐union tissue has only been investigated by two studies, reporting similar expression when compared against BM‐MSC^33^ and healthy cancellous bone.^13^ However, release of Dkk‐1 by atrophic non‐union MSCS cultured in osteogenic conditions was higher than that of BM‐MSCs.^33^ Whilst this study suggests the potential role of Dkk‐1 in the pathophysiology of non‐union, further research is still warranted to better understand the mechanism of action which Dkk‐1 plays in causing non‐union.

There has been emerging evidence over the recent years on the genetic predisposition of fracture non‐union.^19^, ^21^, ^22^, ^23^, ^25^, ^27^, ^28^, ^29^ Numerous genetic polymorphisms associated with fracture non‐union have been identified, with some involving the BMP^25^, ^28^ and MMP pathways.^25^, ^27^ However, most of these studies were significantly underpowered due to is small number of patients and single nucleotide polymorphism (SNP) investigated. Additionally, Wei et al. have identified four micro RNAs (miRNAs) significantly upregulated in atrophic non‐unions (hsa‐miR‐149∗, hsa‐miR‐221, has‐miR‐628‐3p and hsa‐miR‐654‐5p); and result in the significant decrease in the expression of ALPL, PDGFA and BMP2.^9^ Comprehensive analysis on a wider genomic profile combined with bioinformatics may reveal genes, SNPs and miRNAs responsible for the acceleration or inhibition of fracture healing—serving as potential key targets of novel gene therapies.

This literature review is not without its limitations. Firstly, this review excludes animal studies and those which involve experimental animal models, since direct clinical translation is often difficult. Secondly, heterogeneity with the definition of non‐union, timing of tissue harvest and laboratory assays may all account for the different results reported in studies. Lastly, the abbreviation/term MSC is only more recently used in this field, which could be referred to as mesenchymal stem cells or mesenchymal stromal cells.^58^ As such, historical studies using alternative terms such as ‘osteoprogenitors’ and ‘skeletal stem cells’ were excluded as authors felt it does not guarantee the accuracy of comparison made.

There are several strengths of this systematic review. This includes the systematic approach on both screening and analysis of the findings from current literature. Furthermore, this systematic review provides an up‐to‐date understanding on the biological profile of non‐union tissue and relevant tissue at a cellular and molecular level. Due to the huge heterogeneity in available evidence, we are unable to recommend any direct clinical application. The complex pathophysiology of non‐union requires the treating clinician to consider the interaction between biological, physiological and molecular components of the ‘diamond concept’ of bone healing.^59^ Cellular therapies with osteogenic cells and osteoinductive molecules, osteoconductive scaffolds and tissue engineering are treatment strategies which holds great promise.^60^, ^61^ Although still in its early stages, further work on the molecular and genetic profiling of relevant tissue such as patient's serum could serve as an advantageous screening and predictive tool of fracture non‐union.

## 5 CONCLUSION

Fracture non‐union is a challenging condition to treat and poses significant health and socioeconomic burden. Both atrophic and hypertrophic non‐unions were found to possess some degree of vascularity, with resident populations of MSCs with osteogenic capacities. The imbalance in the homeostasis between BMP, chordin, noggin, gremlin and Wnt pathways were believed to be contribute towards non‐union. Increasing body of evidence has identified genetic predisposition in patients with non‐union. Further research is required on determining the sensitivity and specificity of molecular and genetic profiling of relevant tissues as a potential screening biomarker for fracture non‐unions. Other targets of future research include the isolation of specific genes involved in the process of non‐union and the effect of their up‐ or down‐regulation. This along with research around the reactivation of the resident MSCs could potentially revolutionize the management of non‐unions.

## CONFLICTS OF INTEREST

All authors declare no conflict of interest.

## AUTHOR CONTRIBUTION

**Michalis Panteli:** Conceptualization (lead); Data curation (lead); Formal analysis (lead); Investigation (lead); Methodology (lead); Project administration (lead); Resources (lead); Validation (lead); Visualization (lead); Writing – original draft (lead); Writing – review & editing (lead). **James SH Vun:** Data curation (supporting); Formal analysis (supporting); Visualization (supporting); Writing – original draft (supporting); Writing – review & editing (supporting). **Ippokratis Pountos:** Conceptualization (supporting); Methodology (supporting); Supervision (supporting); Visualization (supporting); Writing – original draft (supporting); Writing – review & editing (supporting). **Anthony J Howard:** Data curation (supporting); Formal analysis (supporting); Writing – original draft (supporting); Writing – review & editing (supporting). **Elena Jones:** Conceptualization (supporting); Methodology (supporting); Project administration (supporting); Supervision (supporting); Writing – review & editing (supporting). **P. V. Giannoudis:** Conceptualization (equal); Methodology (equal); Project administration (equal); Supervision (equal); Writing – original draft (supporting); Writing – review & editing (supporting).
